# Use of Tranexamic Acid (TXA) on reducing blood loss during scoliosis surgery in Chinese adolescents

**DOI:** 10.1186/s13013-015-0052-9

**Published:** 2015-10-05

**Authors:** Bobby K. W. Ng, WW Chau, Alec L. H. Hung, Anna CN Hui, Tze Ping Lam, Jack C. Y. Cheng

**Affiliations:** Department of Orthopaedics & Traumatology, Chinese University of Hong Kong, Shatin, Hong Kong; Department of Surgery, Prince of Wales Hospital, Shatin, Hong Kong; Department of Orthopaedics and Traumatology, The Chinese University of Hong Kong, Prince of Wales Hospital, Shatin, N.T. Hong Kong

**Keywords:** Use of tranexamic acid at surgery in AIS

## Abstract

**Background:**

Many reports had been received on the application of antifibrinolytic medications on spinal corrective surgery and the surgical outcome evaluations of its efficacy on reducing blood loss. This study aimed to assess the efficacy of tranexamic acid (TXA) in reducing operative blood loss during posterior spinal fusion for the treatment of severe adolescent idiopathic scoliosis (AIS).

**Methods:**

A retrospective cohort study was carried out on 90 (TXA = 55, Control = 35) AIS girls undergoing posterior spinal surgery. Patients in TXA group used TXA as an antifibrinolytic agent to reduce blood loss, while control group did not. Blood loss, haemoglobin change and amount of blood transfused was estimated from intraoperative measurement by anaesthesiologists. Demographics were compared using Student’s *T*-test or Chi-square test where appropriate. Linear regression modelling was carried out between the use of TXA and total blood loss with controlling of confounding factors.

**Results:**

Mean age and mean maximum major curve were 15.2 and 73°, and 15.3 and 63° in TXA and control groups respectively. TXA group showed significantly less intra-operative blood loss than the control group from intraoperative measurement (1.8 L vs. 3.9 L, *p* < 0.01) and volume of cell saver blood transfused back to patients (0.6 L vs. 1.7 L, *p* < 0.01). TXA group also showed significantly shorter total time taken for surgery (437 min vs. 502 min, *p* < 0.01), and total blood loss per surgical segment level (0.1 L vs. 0.3 L, *p* < 0.01). Regression models showed that the use of TXA decreased total blood loss by 794.3 ml after adjusting for maximum major curve, age, number of segments fused, bone graft, clotting capability, and infusion of coagulation factors.

**Conclusions:**

Patients undergoing posterior spinal corrective surgery with the use of TXA showed much reduced total blood loss, reduced use of transfused blood, much less cell saver blood transfused back to the patient. The total blood loss was decreased by after using TXA after controlling for maximum major curve, age, surgical parameters, clotting capability, and infusion of coagulation factors.

## Background

Reconstructive spinal surgeries have been associated with large blood losses which is a common potential cause of morbidity and could subject to known risk of blood transfusions including blood-borne disease transmission, increased incidence of wound infections, haemolytic and nonhaemolytic transfusion reactions. Despite interventions such as proper patient positioning on the operating room table, pharmaceutical muscle paralysis to decrease intra-abdominal pressure, intraoperative blood salvaging, and controlled hypotensive anaesthesia, blood losses of up to 3 l can still be expected during some frequently performed procedures in spinal surgeries [1–4]. Therefore, any reduction in blood loss and the requirement for patient exposure to homologous blood products would be beneficial.

Since the 1990s, intraoperative administration of antifibrinolytics (tranexamic acid, aprotinin, and e-aminocaproic acid) has gained popularity [5–13]. Antifibrinolytic medications have been applied during cardiac, total joint arthroplasty, and spine surgery with positive effect on blood loss without apparent increase in the risk of postoperative complications as shown by previous studies [7, 10, 12, 14–16]. Tranexamic acid (trans-4-aminomethyl-cyclohexane-1-carboxylic acid, TXA) is a synthetic lysine analogue and exerts its antifibrinolytic effect through the reversible blockade of lysine binding sites on plasminogen molecules. TXA is similar to e-aminocaproic acid but is considered to have 6 to 10 times of the potency *in-vitro* and has been used quite successfully in different surgical procedures to reduce operative blood loss [17].

Reconstructive spinal surgeries have been associated with significant blood loss which is a cause of morbidity. Previous studies reported antifibrinolytic medications could help reduce blood loss during surgery [5, 18, 19]. A recent study on the use of TXA in surgical paediatric patients cohered the results from previous studies on the significant blood loss reduction after using TXA as antifibrolytic agent [20]. However, there is still no report on the possible factors affecting the total blood loss apart from using TXA. The understanding the possible factors, apart from the use of TXA, provides very useful information on other components which could further control the amount of total blood loss at surgery. This study aimed to assess the efficacy of tranexamic acid (TXA) in reducing operative blood loss during posterior spinal fusion for the treatment of severe adolescent idiopathic scoliosis (AIS).

## Methods

A retrospective cohort study was carried out on 90 female adolescents of 10 to 23 years old all undergoing posterior spinal fusion (PSF) procedures at an institution from year 2005 to 2010. They were recruited on a blind selection basis consecutively. Patients’ drug allergy history to TXA was the only contraindication to its use. Otherwise, there was no other contraindication for any patients in receiving TXA. Patients who received intraoperative TXA were categorized into “TXA” group (*N* = 55), and “Control” group for non-receivers (*N* = 35). All patients showed normal blood urea nitrogen, blood creatinine level, bleeding time, prothrombin time (PT), activated partial thrombin time (APTT), international normalized ratio (INR), and serum platelet concentration. Patients with congenital scoliosis or neuromuscular disease or with combined anterior and posterior spinal fusions were excluded. Ethical approval was obtained from the ethics review board of the joint NTEC/CUHK joint-institutional ethics committee.

All patients underwent posterior spinal fusion (PSF) procedures with pedicle screw fixation after general anaesthesia by a skilled anaesthesiologist. The induction and maintenance of the general anaesthesia was accomplished using standard agents. All TXA group patients received an intravenous loading dose of 100 mg/kg TXA followed by a maintenance dose of 10 mg/kg/h, administered until the skin closure. No patient received pre-operative auto pre-donation, intraoperative administration of TXA or other anti-fibrinolytics in the control group. No drain was inserted at the end of the surgical procedure. During the procedures, a special frame which decreased intra-abdominal pressure was used to minimize blood loss. Spinal cord function was monitored by somatosensory- and motor-evoked potentials. All patients received either donated blood or blood from cell saver. Post-operative blood transfusion was given when haemoglobin level dropped below 8 g/dl.

The blood loss was estimated by anaesthesia technician. The intraoperative blood loss was derived by measuring the intraoperative suction drainage and weighing the tapes and sponges. The basic demographic data, surgical statistics, blood loss, clotting capability and infusion of coagulation factors of the two groups were compared by Student’s *T*-test. Sensitivity analysis on regression models was carried out to look for the possible risk factors (clotting capabilities, infusion of coagulation factors) affecting the total blood loss after controlling for confounding factors e.g., age at surgery, maximum major curve, number of segments fused, use of bone graft, length of surgery. Data analysis were carried out by IBM SPSS 20.0 (Armonk, New York). A two-sided *p* value ≤ 0.05 was considered statistically significant.

## Results

This is a retrospective cohort study on 90 (TXA = 55, Control = 35) female patients aiming to look for the effect of the use of tranexamic acid on controlling blood loss in spinal corrective surgery in AIS. There were no significant differences between the TXA and control group in age (15.16 vs. 15.31; *p* = 0.80), mean body weight (Pre-op: 45.25 kg vs. 42.88 kg, *p* = 0.20; Post-op: 44.49 kg vs. 43.39 kg, *p* = 0.65), though the TXA group had a higher pre-operative major curve (73.42 vs. 63.23; *p* = 0.01) (Table 1). TXA group had significantly less intra-operative blood loss (53.0 % reduction) than the control group from the estimation by anaesthesia technician (1.8 L vs. 3.9 L; *p* < 0.01) (Table 2). Patients in the TXA group also received a significantly less volume of salvaged blood from cell saver (0.6 L vs. 1.7 L; *p* < 0.01).

**Table 1:** Baseline characteristics of surgical patients with or without the use of TXA during surgery

Demographics	Tranexamic acid		P
	TXA (*N* = 55)	Control (*N* = 35)	
Age			
Mean ± SD (Range)	15.16 ± 2.61 (10–21)	15.31 ± 2.97 (11–23)	0.80
≥ 12	11 (20.0)	4 (11.4)	0.32
13–14	13 (23.6)	15 (42.9)	
15–16	11 (20.0)	4 (11.4)	
17–18	15 (27.3)	8 (22.9)	
≥ 19	5 (9.1)	4 (11.4)	
Max major curve (Pre-op)	73.42 ± 11.75	63.23 ± 17.20	<0.01
Body weight (kg)			
Pre-op	45.25 ± 8.96	42.88 ± 7.76	0.20
Post-op	44.49 ± 8.14	43.39 ± 8.88	0.65
Armspan (cm)			
Pre-op	157.04 ± 8.99	158.32 ± 9.83	0.54
Post-op	156.04 ± 9.40	158.79 ± 9.23	0.35
Body height (cm)			
Pre-op	154.01 ± 9.33	155.36 ± 7.70	0.48
Post-op	156.83 ± 9.88	157.76 ± 8.82	0.73
BMI (kg/m^2^)			
Pre-op	19.03 ± 3.53	17.76 ± 3.00	0.08
Post-op	18.07 ± 2.77	17.47 ± 3.28	0.48
Number of segments fused	13.51 ± 1.62	12.14 ± 2.79	0.01
Number of pedicle screws inserted	16.15 ± 2.17	16.29 ± 4.34	0.87
Bone graft			
Allograft	1 (2.0)	8 (28.6)	<0.01
Autograft/Both	49 (98.0)	20 (71.4)	
Unknown	5	7	
Length of surgery	436.71 ± 122.53	502.14 ± 85.81	<0.01

*TXA* tranexamic acid used, *Control* none used

**Table 2:** Comparisons on the clotting capabilities, haemoglobin change, blood products transfused, and total blood loss in patients with or without using TXA

Surgical parameters	Tranexamic acid		P
	TXA (*N* = 55)	Control (*N* = 35)	
Clotting capability			
Pre-op PT	11.56 ± 0.87	11.32 ± 1.04	0.24
Pre-op INR	1.07 ± 0.07	1.05 ± 0.06	0.18
Pre-op APTT	37.24 ± 3.88	37.92 ± 4.83	0.46
Haemoglobin change			
Latest postop – preop	−2.99 ± 1.58	−3.33 ± 1.56	0.33
Equivalent volume of whole blood/packed red blood cell transfused			
Intra-operative	472.50 ± 370.48	891.67 ± 358.35	0.04
Post-operative	150.00 ± 256.04	333.33 ± 516.40	0.45
Volume of cell saver blood transfused back to patient (ml)	575.36 ± 412.25	1684.26 ± 1233.91	<0.01
Volume of platelet transfused (ml)	194.41 ± 125.19	219.28 ± 144.17	0.59
Volume of fresh frozen plasma (FFP) transfused (ml)	798.86 ± 520.00	863.18 ± 458.48	0.67
Latest post-operative hemoglobin level	10.00 ± 1.18	9.66 ± 0.75	0.34
Number of days for hemoglobin level reaching stable level post-operatively	4.39 ± 2.12	4.23 ± 1.69	0.80
Total blood loss by anesthetist estimation (ml)	1826.11 ± 1081.45	3889.60 ± 2440.80	<0.01
Total blood loss per segment (ml/segment)	135.62 ± 78.10	328.44 ± 222.68	<0.01

*TXA* tranexamic acid used, *Control* none used, *PT* prothrombin time, *INR* international normalised ratio, *APTT* activated partial thromboplastin time

Different regression models were designed trying to explain the use of TXA on reducing the total blood loss after controlling for different kinds of confounders (Table 3). The statistical significances existed after controlling for possible confounding factors (age at surgery, number of segments fused, maximum major curve, use of bone graft, and length of surgery), without (model 1 to 4) or with (model 5 to 8) the clotting capabilities. The best model (model 6) explained that the use of TXA significantly decreased total blood loss by 794.3 ml (r^2^ = 0.46, B = −5.25, *p* < 0.01) after controlling for maximum major curve and other clinical factors, clotting capabilities, and infusion of coagulation factor. There were no hemodynamic disturbances, apparent thromboembolic complications, or other drug complications associated with its use, such as disturbed colour vision, numbness or weakness, confusion, or allergic reactions major intraoperative complications for any of the treatment groups. No patient had clinical signs of deep venous thrombosis or renal complications.

**Table 3:** Sensitivity analysis on different linear regression models on the use of tranexamic acid, infusion of coagulation factors, and clotting capability on the total blood loss controlled for confounding factors

Models	TXA	Age at surgery	Number of segments fused	Max major curve	Bone graft	Length of surgery	Total FFP transfused	Total platelet transfused	PT (pre-op)	INR (pre-op)	APTT (pre-op)	r^2^	Β	P
1	✓	✓	✓	✓	✓	✓						0.31	−4.98	<0.01
2	✓	✓	✓	✓	✓	✓	✓					0.45	−5.25	<0.01
3	✓	✓	✓	✓	✓	✓		✓				0.44	−5.25	0.05
4	✓	✓	✓	✓	✓	✓	✓	✓				0.27	−3.99	0.14
5	✓	✓	✓	✓	✓	✓			✓	✓	✓	0.32	−5.26	<0.01
6	✓	✓	✓	✓	✓	✓	✓		✓	✓	✓	0.46	−5.25	<0.01
7	✓	✓	✓	✓	✓	✓		✓	✓	✓	✓	0.48	−4.93	0.10
8	✓	✓	✓	✓	✓	✓	✓	✓	✓	✓	✓	0.39	−4.01	0.15

*TXA*, use of tranexamic acid (Yes/No), *FFP* fresh frozen plasma, *PT* prothrombin time, *INR* international normalised ratio, *APTT* activated partial thromboplastin time

## Discussion

Pharmacological therapies are commonly used to reduce blood loss and blood transfusions in surgery. Tranexamic acid or TXA was previously shown to be as effective as other antifibrinolytics but at a much lower cost [21, 22]. The safety and efficacy of TXA are still controversial probably due to the difference in the dose of TXA used and type of surgery [21, 23]. In this retrospective study, it was shown that TXA could significantly reduce blood loss with up to 53.0 % reduction which is in concordance with some similar studies [19, 24, 25]. Intraoperative estimated blood loss was shown to be decreased by 56.8 % for patients using TXA, reported from a comparative analysis of 106 consecutive adolescents undergoing PSF in Japan [14]. Moreover, contributing factors that could alter blood loss were included in regression analysis, thus proving the efficacy of TXA in reducing blood loss even after adjustment with possible confounding factors, clotting capabilities and infusion of coagulation factors. All the patients in this study showed no severe complications which demonstrated the safety of TXA.

The use of TXA in major paediatric surgery was proven to be better than the other commonly used antifibrinolytic agents e.g., aprotinin and aminocaproic acid [20, 21, 23, 26]. Results from a recent randomized double-blinded pilot study studying the efficacy of aminocaproic acid versus TXA in paediatric spinal deformity surgery concluded that TXA was associated with a lower allogenic transfusion requirement, less alteration in postoperative clotting studies, and a trend toward lower blood loss in paediatric posterior spinal fusion patients [24]. The cost of TXA is higher than aminocaproic acid, therefore, the use of TXA is excellent for long and complex surgeries e.g., scoliosis and surgeries with the use of autograft and long fusion levels [27]. Aprotinin was associated with severe side effects e.g., myocardial infraction, heart failure, and renal failure [28–31], although it was effective in reducing total blood loss after paediatric cardiac surgery [32]. Higher costs when using aprotinin make TXA a better option with similar efficacy. In this study, we clearly proved that the use of TXA in surgical scoliotic cases greatly reduced the volume of whole blood and blood from cell saver transfused back to the patients, although no comparison on the use of other antifibrinolytic agents were carried out because of the standard and consistency of clinical practice.

Studies tried to look for the factors other than the use of TXA affecting the total blood loss, however, no conclusion has been clearly made probably as a result of limited data collected for data analysis. Discussions on autologous blood transfusion and reinfusion of salvaged blood from cell saver machine being the causative factor have been introduced for over a decade [6, 20, 25]. Transfusion requirements and coagulation parameters were first introduced by Sethna et al. in a study with 44 children and adolescents undergoing scoliotic surgical corrections through comparative analyses [5]. Other factors like age, gender, and number of vertebral levels fused, were introduced in recent publications [20, 25]. Length of hospital stay was discussed in a couple of reports, while this was the secondary clinical outcome of the use of TXA instead of being a factor on controlling total blood loss [33, 34]. At the ever-vigorous surgical environment, there are many factors which can affect the total blood loss, not solely dictated by the use of TXA, and these have to be controlled for us to draw a conclusion on the effect of TXA on controlling blood loss at surgery. This study collected information in possible confounding factors on total blood loss with the use of TXA at surgery in Chinese AIS patients, and confirmed the efficacy of TXA after controlling major confounding factors.

In this study, total blood loss estimated by anaesthesia technician was significantly reduced in patients using TXA at surgery. At the same time, volume of blood transfused from cell saver was also much reduced in TXA group. A recent study on the use of TXA in 49 surgical AIS patients undergoing posterior spinal fusion by a single surgeon showed similar findings on great decrease in total blood loss as of the present study [20]. The volume of blood transfused, however, did not appear to be affected [20]. In this study, the volume of salvaged blood from cell saver was much decreased in TXA group. Nonetheless, the total amount of blood loss and cell saver blood returned to the patients were relatively huge. These were because the spinal curvatures were large and the fusion levels were high. Similar results were obtained from surgeries on severe curvatures [19, 33]. In summary. we have established a very tight transfusion protocol for AIS surgery, which makes the results different from the study from Lykissas and colleagues [20]. A recent randomized control trial was carried out to look for the efficacy of different antifibrinolytics in AIS. TXA and epsilon-aminocaproic acid effectively reduced estimated and actual blood loss, as well as declined in haematocrit after surgery compared with saline solution [25].

The present study also gives us an idea on the rate of blood loss decrease after using TXA at surgery with proper adjustment of confounding factors. Regarding to the transfusion rate being another important parameter evaluating the efficacy of antifibrinolytics, authors, the factors affecting the transfusion rate is mainly a surgeon-decisive issue, namely patient comorbidities, patient preference, preoperative blood donation, and clinical judgment [25]. Similar findings were found in another RCT published in Cochrane Library [35]. A systematic review and meta-analysis on the use of intravenous TXA in spinal surgery carried out in China showed much reduced volume of blood loss and volume of transfused packed cells, although patient age was one of the study exclusion criteria making the analyses not totally applicable to present study [26]. An extended multi-centre review on the use of different antifibrinolytic agents to reduce blood loss during vertebral column resection in paediatrics also confirmed the effectiveness of reduced estimated blood loss when using TXA during surgery after normalized to patient size and levels excised [19]. Similar observations are well-documented in many previous studies [5, 6, 15, 16, 18, 19, 33–39]. The present study is the first study on the evaluation of efficacy of TXA in Chinese AIS patients, with the novel findings of factors affecting total blood loss.

This is the first report on the use of TXA in surgical Chinese AIS patients. The factors affecting the total blood loss are derived by a series of sensitivity analysis through different regression models with the proper controlling of confounding factors.

### Limitations

There are limitations we have to consider in this study. The small sample size and retrospective nature of this comparative study would limit the data generalizability. Our surgeons and anaesthetists had many years of experience in spinal surgery before beginning of this study, the ever-improving surgical skills over the years could influence the amount of total blood loss. The concern on the effect of the improvement of surgical skills on the amount of total blood loss was proven minimal after a closed monitoring of difference of total surgical blood loss over the study period. Yet a prospective dose-ranging study is still required to determine the optimal dose for spine surgery on patients with idiopathic scoliosis. To provide additional information on the efficacy and safety of TXA, multi-centre randomized prospective analysis in the future is warranted.

## Conclusions

Patients undergoing posterior spinal corrective surgery with the use of TXA showed much reduced total blood loss, reduced use of transfused blood, and much less cell saver blood transfused back to the patient. Using TXA at surgery, the total blood loss was decreased by 794.3 ml after controlling for maximum major curve and other clinical factors, clotting capabilities, and infusion of coagulation factor. Use of tranexamic acid significantly reduced total surgical blood loss over corrective surgery for severe AIS patients, after adjusting for possible confounding factors, clotting capabilities, and infusion of coagulation factors.
